# Hospitalization and Mortality Risk for COVID-19 Cases With SARS-CoV-2 AY.4.2 (VUI-21OCT-01) Compared to Non-AY.4.2 Delta Variant Sublineages^[Author-notes jiac063-FM1]^

**DOI:** 10.1093/infdis/jiac063

**Published:** 2022-02-20

**Authors:** Tommy Nyberg, Katie Harman, Asad Zaidi, Shaun R Seaman, Nick Andrews, Sophie G Nash, Andre Charlett, Jamie Lopez Bernal, Richard Myers, Natalie Groves, Eileen Gallagher, Saheer Gharbia, Meera Chand, Simon Thelwall, Daniela De Angelis, Gavin Dabrera, Anne M Presanis

**Affiliations:** Medical Research Council Biostatistics Unit, University of Cambridge, Cambridge, United Kingdom; COVID-19 National Epidemiology Cell, UK Health Security Agency, London, United Kingdom; COVID-19 National Epidemiology Cell, UK Health Security Agency, London, United Kingdom; Medical Research Council Biostatistics Unit, University of Cambridge, Cambridge, United Kingdom; Immunisation and Countermeasures Division, UK Health Security Agency, London, United Kingdom; COVID-19 National Epidemiology Cell, UK Health Security Agency, London, United Kingdom; National Infection Service, UK Health Security Agency, London, United Kingdom; Immunisation and Countermeasures Division, UK Health Security Agency, London, United Kingdom; Genomics Cell, UK Health Security Agency, London, United Kingdom; Genomics Cell, UK Health Security Agency, London, United Kingdom; Genomics Cell, UK Health Security Agency, London, United Kingdom; Genomics Programme, UK Health Security Agency, London, United Kingdom; Genomics Cell, UK Health Security Agency, London, United Kingdom; COVID-19 National Epidemiology Cell, UK Health Security Agency, London, United Kingdom; Medical Research Council Biostatistics Unit, University of Cambridge, Cambridge, United Kingdom; COVID-19 National Epidemiology Cell, UK Health Security Agency, London, United Kingdom; Medical Research Council Biostatistics Unit, University of Cambridge, Cambridge, United Kingdom

**Keywords:** COVID-19, SARS-CoV-2, AY.4.2, VUI-21OCT-01, hospitalization, mortality

## Abstract

To investigate if the AY.4.2 sublineage of the SARS-CoV-2 delta variant is associated with hospitalization and mortality risks that differ from non-AY.4.2 delta risks, we performed a retrospective cohort study of sequencing-confirmed COVID-19 cases in England based on linkage of routine health care datasets. Using stratified Cox regression, we estimated adjusted hazard ratios (aHR) of hospital admission (aHR = 0.85; 95% confidence interval [CI], .77–.94), hospital admission or emergency care attendance (aHR = 0.87; 95% CI, .81–.94), and COVID-19 mortality (aHR = 0.85; 95% CI, .71–1.03). The results indicate that the risks of hospitalization and mortality are similar or lower for AY.4.2 compared to cases with other delta sublineages.

## Teaser

COVID-19 cases with the AY.4.2 sublineage of the delta variant had lower or similar risks of hospital admission, hospital admission or emergency care attendance, and COVID-19 mortality compared to cases with other delta variant sublineages.

A new severe acute respiratory syndrome coronavirus 2 (SARS-CoV-2) delta (Pango lineage B.1.617.2; https://cov-lineages.org) variant sublineage, AY.4.2, slowly increased in prevalence among coronavirus disease 2019 (COVID-19) cases in England, from <0.01% in early June to 20.3% in the week commencing 15 November 2021 [1]. After preliminary analyses suggested that AY.4.2 might have a small transmission advantage compared to non-AY.4.2 delta [1], AY.4.2 was designated a variant under investigation (VUI-21OCT-01) by the UK Health Security Agency (UKHSA) on 20 October 2021 [1]. It is unknown whether AY.4.2 is associated with a differently severe COVID-19 than non-AY.4.2 delta. We therefore investigated the relative severity of AY.4.2 compared to other delta cases using a retrospective cohort study.

## METHODS

The study population comprised COVID-19 cases in England with a first positive specimen between 21 June and 7 November 2021 who were infected with AY.4.2 or a non-AY.4.2 delta variant based on whole-genome sequencing. Data on these cases were linked to national hospital care and mortality datasets on 1 December 2021. Before the week commencing 21 June, <0.2% of sequencing-confirmed delta cases had the AY.4.2 sublineage [1]; during the inclusion period the prevalence of AY.4.2 among new sequencing-confirmed cases increased from 0.2% to 15% (Supplementary Figure 1). The data linkage, inclusion criteria, outcome and confounder data sources and definitions, and the analysis strategy have been described in a recent article [2].

Using stratified Cox regression models, we estimated hazard ratios (HRs) of hospital admission and hospital admission or emergency care attendance within 14 days, and of COVID-19 or all-cause mortality within 28 days after a first positive COVID-19 test. These models were stratified for week of specimen and lower tier local authority of residence, to account for reporting delays and unobserved confounders that may differ by calendar time and locality. Regression adjustment was used for age and index of multiple deprivation rank (each modelled using restricted cubic splines with 4 knots), date of specimen (linear term), sex, ethnicity, vaccination status, and recent international travel. We additionally estimated the HRs within subgroups based on symptom or vaccination status. In supplementary analyses, we explored the sensitivity of the HRs to alternative adjustment approaches and to bias due to differences of epidemic phase of the sublineages [3].

### Ethics

This surveillance was performed as part of UKHSA's responsibility to monitor COVID-19 during the current pandemic. UKHSA has legal permission, provided by Regulation 3 of The Health Service (Control of Patient Information) Regulations 2002 to process confidential patient information under Sections 3(i) (a) to (c), 3(i)(d) (i) and (ii) and 3(iii) as part of its outbreak response activities. This study falls within the research activities approved by the UKHSA Research Ethics and Governance Group.

## RESULTS

### Characteristics

A total of 28 736 AY.4.2 cases and 492 301 non-AY.4.2 delta cases were identified through the data linkage and included in the study. The age distribution was similar between AY.4.2 cases (median 31 years, interquartile range 13–48) and non-AY.4.2 delta cases (median 30 years, interquartile range 15–48). A slightly greater proportion of AY.4.2 cases than non-AY.4.2 delta cases resided in South-East England and in less-deprived areas. As expected, the AY.4.2 cases tended to have tested positive in more recent weeks (Supplementary Table 1).

### Hospitalization and Mortality

After adjustment for confounders, the risks of hospital admission (HR = 0.85; 95% confidence interval [CI], .77–.94) and hospital admission or emergency care attendance (HR = 0.87; 95% CI, .81–.94) were lower for AY.4.2 compared to non-AY.4.2 delta cases. There was no significant difference in the risk of COVID-19 mortality (HR = 0.85; 95% CI, .71–1.03) but the risk of all-cause mortality was lower (HR = 0.82; 95% CI, .69–.98) for AY.4.2 compared to non-AY.4.2 delta variant cases (Table 1). For the outcome hospital admission, the difference in risk was somewhat more pronounced for unvaccinated AY.4.2 versus non-AY.4.2 cases (HR = 0.79; 95% CI, .65–.95) than for vaccinated AY.4.2 versus non-AY.4.2 cases (HR = 0.89; 95% CI, .79–1.01); otherwise, the results for AY.4.2 versus non-AY.4.2 delta cases were similar in the subgroups defined by vaccination status or symptom status (Table 2). The sensitivity analysis exploring alternative adjustment approaches yielded HRs similar to those from the primary analysis (Supplementary Table 2). The sensitivity analysis adjusting for epidemic phase bias considered multiple scenarios, which suggested that the risks of all considered COVID-19 severity outcomes might be slightly lower for AY.4.2 compared to non-AY.4.2 delta cases (Supplementary Tables 3 and 4), consistent with the primary analysis.

**Table 1.** Hospitalization and Mortality Outcomes for COVID-19 Cases Infected With AY.4.2 Compared to Non-AY.4.2 Delta Variants

Outcome	AY.4.2, n/N (%)	Non-AY.4.2 Delta, n/N (%)	HR (95% CI), AY.4.2 vs Non-AY.4.2 Delta	
			Unadjusted	Adjusted^a^
Hospital admission within 14 d after specimen	415/28736 (1.4)	10766/492301 (2.2)	0.66 (.60–.72)	0.85 (.77–.94)
Hospital admission or emergency care attendance within 14 d after specimen	847/28736 (2.9)	19808/492301 (4.0)	0.73 (.68–.78)	0.87 (.81–.94)
COVID-19 death within 28 d after specimen	143/28736 (0.50)	3465/492301 (0.70)	0.71 (.60–.84)	0.85 (.71–1.03)
Death due to any cause within 28 d after specimen	165/28736 (0.57)	3940/492301 (0.80)	0.72 (.61–.84)	0.82 (.69–.98)

Stratification for week of specimen and lower tier local authority of residence; regression adjustment for date of specimen (linear), age (restricted cubic splines with 4 knots), index of multiple deprivation rank (restricted cubic splines with 4 knots), sex, ethnicity (white, Asian, black, other/mixed/unknown), vaccination status at date of positive test (unvaccinated, <21 days since first dose, ≥21 days since first dose and < 14 days since second dose, ≥14 days since second dose) and international travel within 14 days before positive test.

**Table 2.** Hospitalization and Mortality Outcomes for COVID-19 Cases Infected With AY.4.2 Compared to Non-AY.4.2 Delta Variants, by Subgroups

Outcome	AY.4.2, n/N (%)	Non-AY.4.2 Delta, n/N (%)	HR (95% CI), AY.4.2 vs Non-AY.4.2 Delta	
			Unadjusted	Adjusted^a^
**Symptomatic or likely symptomatic cases** ^b^				
Hospital admission within 14 d after specimen	415/16305 (2.5)	10766/288776 (3.7)	0.68 (.61–.75)	0.89 (.80–.98)
Hospital admission or emergency care attendance within 14 d after specimen	847/16305 (5.2)	19808/288776 (6.9)	0.75 (.70–.80)	0.90 (.84–.97)
COVID-19 death within 28 d after specimen	143/16305 (0.88)	3465/288776 (1.2)	0.73 (.62–.86)	0.97 (.80–1.17)
Death due to any cause within 28 d after specimen	161/16305 (0.99)	3886/288776 (1.3)	0.73 (.63–.86)	0.92 (.76–1.10)
**Unvaccinated or with <21 d since first vaccine dose**				
Hospital admission within 14 d after specimen	128/13520 (0.95)	4310/235971 (1.8)	0.52 (.43–.61)	0.79 (.65–.95)
Hospital admission or emergency care attendance within 14 d after specimen	326/13520 (2.4)	8807/235971 (3.7)	0.64 (.57–.72)	0.90 (.80–1.01)
COVID-19 death within 28 d after specimen	26/13520 (0.19)	819/235971 (0.35)	0.55 (.38–.82)	0.77 (.46–1.30)
Death due to any cause within 28 d after specimen	27/13520 (0.20)	896/235971 (0.38)	0.53 (.36–.77)	0.67 (.40–1.10)
**≥21 d since first vaccine dose (with or without a second vaccine dose)**				
Hospital admission within 14 d after specimen	287/15216 (1.9)	6456/256330 (2.5)	0.75 (.66–.84)	0.89 (.79–1.01)
Hospital admission or emergency care attendance within 14 d after specimen	521/15216 (3.4)	11001/256330 (4.3)	0.79 (.73–.87)	0.87 (.79–.95)
COVID-19 death within 28 d after specimen	117/15216 (0.77)	2646/256330 (1.0)	0.75 (.62–.90)	0.89 (.72–1.10)
Death due to any cause within 28 d after specimen	138/15216 (0.91)	3044/256330 (1.2)	0.76 (.64–.91)	0.86 (.71–1.04)
**≥14 d since second vaccine dose**				
Hospital admission within 14 d after specimen	267/13341 (2.0)	5928/201625 (2.9)	0.68 (.60–.76)	0.87 (.76–.99)
Hospital admission or emergency care attendance within 14 d after specimen	475/13341 (3.6)	9674/201625 (4.8)	0.74 (.67–.81)	0.85 (.78–.94)
COVID-19 death within 28 d after specimen	113/13341 (0. 85)	2535/201625 (1.3)	0.67 (.56–.81)	0.88 (.71–1.10)
Death due to any cause within 28 d after specimen	133/13341 (1.0)	2916/201625 (1.4)	0.69 (.58–.82)	0.85 (.70–1.03)

Stratification for week of specimen and lower tier local authority of residence; regression adjustment for date of specimen (linear), age (restricted cubic splines with 4 knots), index of multiple deprivation rank (restricted cubic splines with 4 knots), sex, ethnicity (white, Asian, black, other/mixed/unknown), vaccination status at date of positive test (unvaccinated, <21 days since first dose, ≥21 days since first dose and < 14 days since second dose, ≥14 days since second dose) and international travel within 14 days before positive test.

Cases who (1) were recorded to be symptomatic at the time of positive test, (2) were hospitalized, attended emergency care, or died with COVID-19 mentioned on the death certificate, or (3) were tested through the pillar 1 hospital testing program. See Supplementary Material for a justification of this definition.

## DISCUSSION

Based on record linkage of sequencing-confirmed COVID-19 cases in England, we found that the risks of hospitalization and mortality outcomes were similar or lower for cases infected with the AY.4.2 compared to non-AY.4.2 sublineages of the delta variant of SARS-CoV-2. The results were similar when restricted to symptomatic and likely symptomatic cases, or to vaccinated or unvaccinated subgroups, or after additional adjustment for time since second vaccine dose. Further sensitivity analyses to adjust for the effect of epidemic phase bias [3] consistently suggested that the risks of the hospitalization outcomes are similar or lower for AY.4.2 than non-AY.4.2 delta cases.

Strengths of this analysis include the use of timely population datasets that cover all hospitalization events and deaths for COVID-19 cases in England. Limitations include reporting delays of the outcome events, which may differ over time and by hospital trust. However, after stratification for calendar period and area, the reporting delays should not differ systematically by sublineage. A further limitation is the restriction to cases confirmed through sequencing, due to a lack of other methods capable of distinguishing different delta sublineages. During the study period, the median daily sequencing coverage of new COVID-19 cases was 16.5% (range, 6.5%–27.2%) [1]. More severe cases with higher viral loads may be preferentially selected for sequencing, which may restrict the generalizability of the findings. However, similar cycle threshold counts were reported between individuals infected with AY.4.2 or non-AY.4.2 identified in the REACT-1 random testing survey [4]. Hence, there is no reason to expect that such selection differed systematically by sublineage.

Several variants of the SARS-CoV-2 virus have evolved during the COVID-19 pandemic of 2020–2022. In England, the alpha (Pango lineage B.1.1.7) variant was detected in November 2020 and was found to be associated with higher transmissibility [5], and higher risks of hospital admission [6, 7] and mortality [6, 8], than previously circulating wild-type SARS-CoV-2. In March 2021, the delta variant was detected in England and soon became the dominant variant in the country. Delta is associated with higher transmissibility [9], partial vaccine escape [10, 11], and higher risk of hospitalization [2, 10, 12] and mortality [12], compared to the alpha variant. Recently, cases with AY.4.2 were reported to be less likely to experience symptomatic disease than cases with other delta sublineages [4]. Although our results indicated similar proportions with symptomatic disease in AY.4.2 and non-AY.4.2 cases, a lower propensity to cause symptomatic disease is consistent with the findings of lower severity risk with AY.4.2. Some preliminary analyses suggest that AY.4.2 might have a small transmission advantage, with 15% higher growth rate and reproduction number compared to non-AY.4.2 delta [1, 13]. Preliminary analyses also suggest that the available vaccines are equally efficient [14] and equally effective against symptomatic disease and hospitalization [1] for AY.4.2 as for non-AY.4.2 delta sublineages. In line with this lack of difference in vaccine effectiveness, and in contrast to findings for the previous new more transmissible variants, our results suggest that the risk of severe disease is lower or similar for cases with the AY.4.2 sublineage compared to that for cases with other delta variants. More recently, the omicron (Pango lineage B.1.1.529) variant has become dominant in England and much of the world. Omicron has been found to be associated with lower hospitalization and mortality risks than delta [15]. Similar to the emergence of the AY.4.2 delta sublineage, an omicron sublineage (BA.2) with a potential transmission advantage has recently been identified [1]. The findings in our study highlight the importance of assessing severity differences between SARS-CoV-2 variant sublineages, and provide a baseline for future research on the relative severity between delta or delta variant sublineages and other circulating variants such as omicron and its sublineages.

## Supplementary Data

Supplementary materials are available at *The Journal of Infectious Diseases* online. Supplementary materials consist of data provided by the author that are published to benefit the reader. The posted materials are not copyedited. The contents of all supplementary data are the sole responsibility of the authors. Questions or messages regarding errors should be addressed to the author.

jiac063_suppl_Supplementary_Material

## Notes

***Author contributions*.** T. N., K. H., A. Z., S. R. S., N. A., S. G. N., A. C., S. T., D. D. A., G. D., and A. M. P. contributed to the design of the study. K. H., A. Z., S. G. N., A. C., J. L. B., R. M., N. G., E. G., S. G., M. C., S. T., and G. D. contributed to data collection and creation of data resources. T. N. performed the statistical analysis, with support from S. R. S., D. D. A., and A. M. P. Funding was acquired by A. M. P., A. C., and D. D. A. The first draft of the manuscript was written by T. N., and A. M. P. supwriting. T. N., K. H., A. Z., S. R. S., S. G. N., A. C., S. T., D. D. A., G. D., and A. M. P. contributed to the writing of a revised draft. All authors critically reviewed and edited the final manuscript. All authors approved the final version of the manuscript and agree to be accountable for the work.

***Disclaimer***. The funders played no direct role in the study. The views expressed are those of the authors and not necessarily those of the NIHR or the Department of Health and Social Care.

***Financial support.*** This work was supported by the United Kingdom Research and Innovation Medical Research Council (UKRI MRC) (unit programme numbers MC_UU_00002/11 to D. D. A. and A. M. P., and MC_UU_00002/10 to S. R. S.); UKRI MRC/ Department of Health and Social Care National Institute for Health Research (NIHR) COVID-19 Rapid Response Call (grant number MC_PC_19074 to T. N., A. C., D. D. A., and A. M. P.); NIHR Health Protection Unit in Behavioural Science and Evaluation (to D. D. A.); and NIHR Cambridge Biomedical Research Centre (grant number BRC-1215-20014).
