# Supplementary material for: Hospitalisation and mortality risk for COVID-19 cases with SARS-CoV-2 AY.4.2 (VUI-21OCT-01) compared to non-AY.4.2 Delta variant sub-lineages
Source: J Infect Dis. 2022 Feb 20:jiac063. doi: 10.1093/infdis/jiac063 (PMC8903446; doi:10.1093/infdis/jiac063)
Supplement: Supplementary file 1 [file jiac063_suppl_supplementary_material.docx]

Hospitalisation and mortality risk for COVID-19 cases with SARS-CoV-2 AY.4.2 (VUI-21OCT-01) compared to non-AY.4.2 Delta variant sub-lineages

Supplementary material 

**Sequencing-confirmed SARS-CoV-2 variants in new cases during the study period**

Supplementary Figure 1 shows the distribution of sequencing-confirmed SARS-CoV-2 variants in new cases in England during the study period between 21 June and 7 November 2021, by calendar week. The Delta variant (B.1.617.2) was the dominant variant with few cases being infected with other variants. The AY.4.2 sub-lineage of the Delta variant increased in prevalence from 0.2% in the first week to 15% in the final week of the inclusion period.

**Supplementary Figure 1:** Distribution of sequencing-confirmed SARS-CoV-2 variants in new COVID-19 cases between 21 June and 7 November 2021 whose specimens were assessed by whole genome sequencing. 

**Characteristics**

Supplementary Table 1 shows characteristics of the study population.

**Supplementary Table 1:** Characteristics.

| **Characteristic** |  | **Overall** | **Non-AY.4.2 Delta** | **AY.4.2** |
| --- | --- | --- | --- | --- |
|  |  | **n (%)** | **n (%)** | **n (%)** |
| Total |  | 521037 | 492301 | 28736 |
| Age |  |  |  |  |
|  | 0-9 | 43691 (8.4%) | 40783 (8.3%) | 2908 (10.1%) |
|  | 10-19 | 130174 (25.0%) | 121851 (24.8%) | 8323 (29.0%) |
|  | 20-29 | 81692 (15.7%) | 78992 (16.0%) | 2700 (9.4%) |
|  | 30-39 | 73196 (14.0%) | 69716 (14.2%) | 3480 (12.1%) |
|  | 40-49 | 74481 (14.3%) | 69892 (14.2%) | 4589 (16.0%) |
|  | 50-59 | 57363 (11.0%) | 54009 (11.0%) | 3354 (11.7%) |
|  | 60-69 | 31355 (6.0%) | 29502 (6.0%) | 1853 (6.4%) |
|  | 70-79 | 18726 (3.6%) | 17669 (3.6%) | 1057 (3.7%) |
|  | ≥80 | 10359 (2.0%) | 9887 (2.0%) | 472 (1.6%) |
| Sex |  |  |  |  |
|  | Female | 269929 (51.8%) | 254674 (51.7%) | 15255 (53.1%) |
|  | Male | 251108 (48.2%) | 237627 (48.3%) | 13481 (46.9%) |
| Ethnicity |  |  |  |  |
|  | White | 435762 (83.6%) | 411374 (83.6%) | 24388 (84.9%) |
|  | Asian | 38523 (7.4%) | 36463 (7.4%) | 2060 (7.2%) |
|  | Black | 15855 (3.0%) | 15087 (3.1%) | 768 (2.7%) |
|  | Mixed/Other/Unknown | 30897 (5.9%) | 29377 (6.0%) | 1520 (5.3%) |
| Region of residence |  |  |  |  |
|  | London | 65138 (12.5%) | 61740 (12.5%) | 3398 (11.8%) |
|  | East Midlands | 41377 (7.9%) | 39402 (8.0%) | 1975 (6.9%) |
|  | East of England | 59322 (11.4%) | 56051 (11.4%) | 3271 (11.4%) |
|  | North East | 25434 (4.9%) | 24739 (5.0%) | 695 (2.4%) |
|  | North West | 71465 (13.7%) | 68355 (13.9%) | 3110 (10.8%) |
|  | South East | 76305 (14.6%) | 70765 (14.4%) | 5540 (19.3%) |
|  | South West | 62868 (12.1%) | 58922 (12.0%) | 3946 (13.7%) |
|  | West Midlands | 55628 (10.7%) | 51717 (10.5%) | 3911 (13.6%) |
|  | Yorkshire and Humber | 63500 (12.2%) | 60610 (12.3%) | 2890 (10.1%) |
| Index of multiple deprivation |  |  |  |  |
|  | 1st quintile (most deprived) | 100524 (19.3%) | 95996 (19.5%) | 4528 (15.8%) |
|  | 2nd quintile | 101035 (19.4%) | 95828 (19.5%) | 5207 (18.1%) |
|  | 3rd quintile | 104190 (20.0%) | 98321 (20.0%) | 5869 (20.4%) |
|  | 4th quintile | 106356 (20.4%) | 100104 (20.3%) | 6252 (21.8%) |
|  | 5th quintile (least deprived) | 108932 (20.9%) | 102052 (20.7%) | 6880 (23.9%) |
| Calendar week of specimen |  |  |  |  |
|  | 25-26 | 39064 (7.5%) | 38979 (7.9%) | 85 (0.3%) |
|  | 27-28 | 36851 (7.1%) | 36587 (7.4%) | 264 (0.9%) |
|  | 29-30 | 50647 (9.7%) | 50110 (10.2%) | 537 (1.9%) |
|  | 31-32 | 50593 (9.7%) | 49728 (10.1%) | 865 (3.0%) |
|  | 33-34 | 55770 (10.7%) | 54152 (11.0%) | 1618 (5.6%) |
|  | 35-36 | 53455 (10.3%) | 51407 (10.4%) | 2048 (7.1%) |
|  | 37-38 | 54405 (10.4%) | 51012 (10.4%) | 3393 (11.8%) |
|  | 39-40 | 58236 (11.2%) | 53468 (10.9%) | 4768 (16.6%) |
|  | 41-42 | 57971 (11.1%) | 51798 (10.5%) | 6173 (21.5%) |
|  | 43-44 | 64045 (12.3%) | 55060 (11.2%) | 8985 (31.3%) |
| Vaccination status at date of specimen ^a^ |  |  |  |  |
|  | Unvaccinated | 231204 (44.4%) | 218361 (44.4%) | 12843 (44.7%) |
|  | <21 days after first vaccination dose | 18287 (3.5%) | 17610 (3.6%) | 677 (2.4%) |
|  | ≥21 days after first vaccination dose ^b^ | 56580 (10.9%) | 54705 (11.1%) | 1875 (6.5%) |
|  | 14-174 days after second vaccination dose^ c^ | 184900 (35.5%) | 174440 (35.4%) | 10460 (36.4%) |
|  | ≥175 days after second vaccination dose^ c^ | 30066 (5.8%) | 27185 (5.5%) | 2881 (10.0%) |
| Recent international travel within 14 days before specimen |  |  |  |  |
|  | No known travel | 497518 (95.5%) | 470004 (95.5%) | 27514 (95.7%) |
|  | Yes | 23519 (4.5%) | 22297 (4.5%) | 1222 (4.3%) |
| Symptom status |  |  |  |  |
|  | Asymptomatic | 209629 (40.2%) | 197622 (40.1%) | 12007 (41.8%) |
|  | Symptomatic | 246417 (47.3%) | 232270 (47.2%) | 14147 (49.2%) |
|  | Unknown | 64991 (12.5%) | 62409 (12.7%) | 2582 (9.0%) |

^a^ The frequencies for cases with two doses include 5282 cases who had received a third vaccine dose before positive specimen, 1460 of whom had at least 14 days between the third dose and positive specimen.
^b^ Among cases with ≥21 days since their first vaccine dose, 9633 cases had received AstraZeneca, 43001 cases had received Pfizer, 3910 cases had received Moderna, and 36 cases had received other vaccines.
^c^ Among cases with ≥14 days since their second dose, 150115 had received two doses of AstraZeneca, 62657 had received two doses of Pfizer, 1510 had received two doses of Moderna, 397 had received combinations of the above three vaccines, and 287 cases had received at least one dose of another vaccine.

**Sensitivity analysis: alternative stratification and adjustment strategies**

**Methods**

To assess the sensitivity of the results to the primary adjustment approach and which variables were chosen for stratification or regression adjustments, we performed the following sensitivity analyses: 

1. Instead of stratifying for lower tier local authority (LTLA), we stratified for the broader area level variables upper tier local authority (UTLA) or region. 

2. Instead of calendar week, we stratified for exact calendar date or fortnight. 

3. Instead of stratifying only for calendar week and LTLA, we additionally stratified for age group and vaccination status instead of using these variables for regression adjustments as in the primary model.

4. Instead of using stratification to adjust for calendar week and LTLA, we used no stratification and included all adjustment variable as regression covariates, with LTLA included as a random effect.

5. Instead of adjusting for vaccination status in four categories, we additionally adjusted for time since second vaccine dose in the intervals 14-55, 56-97, 98-139, 140-174 or ≥175 days since second dose. We then repeated this analysis, excluding cases who had received a third vaccine dose.

**Results**

Supplementary Table 2 shows the results of these sensitivity analyses. For all considered outcomes, all of these analyses resulted in estimates of the HRs for AY.4.2 compared to non-AY.4.2 Delta cases that were similar to those from the primary analysis. For both mortality outcomes, the 95% CIs included 1.0 when using a finer stratification for exact calendar date or the additional adjustment variables age group and vaccination status. Adjustment for time since second vaccine dose did not substantially change the HRs.

**Supplementary Table 2:** Sensitivity analyses to assess the impact of stratification or regression adjustments for alternative or additional variables.

| **Sensitivity analysis** | **HR (95% CI), AY.4.2 vs non-AY.4.2 Delta** ^**a**^ |  |  |  |
| --- | --- | --- | --- | --- |
|  | **Hospital admission within 14 days after specimen** | **Hospital admission or emergency care attendance within 14 days after specimen** | **COVID-19 death within 28 days after specimen** | **Death due to any cause within 28 days after specimen** |
| Primary model | 0.85 (0.77-0.94) | 0.87 (0.81-0.94) | 0.85 (0.71-1.03) | 0.82 (0.69-0.98) |
| Stratification for UTLA instead of LTLA | 0.84 (0.76-0.93) | 0.87 (0.81-0.94) | 0.85 (0.71-1.02) | 0.83 (0.70-0.98) |
| Stratification for region instead of LTLA | 0.84 (0.76-0.93) | 0.88 (0.82-0.94) | 0.85 (0.72-1.01) | 0.84 (0.72-0.98) |
| Stratification for exact calendar date instead of week | 0.88 (0.79-0.99) | 0.88 (0.82-0.95) | 0.91 (0.72-1.15) | 0.84 (0.68-1.05) |
| Stratification for fortnight instead of week | 0.85 (0.77-0.94) | 0.87 (0.81-0.94) | 0.82 (0.69-0.99) | 0.80 (0.68-0.95) |
| Stratification for week, LTLA, age group and vaccination status | 0.86 (0.76-0.96) | 0.86 (0.79-0.94) | 0.87 (0.70-1.09) | 0.82 (0.67-1.01) |
| No stratification; regression adjustment for all variables (LTLA included as random effect) | 0.85 (0.77-0.94) | 0.88 (0.82-0.94) | 0.86 (0.72-1.02) | 0.84 (0.72-0.99) |
| Additional regression adjustment for time since second vaccine dose ^b^ | 0.84 (0.76-0.94) | 0.87 (0.81-0.94) | 0.85 (0.70-1.02) | 0.81 (0.68-0.97) |
| Additional regression adjustment for time since second vaccine dose, excluding cases with three vaccine doses | 0.86 (0.77-0.95) | 0.87 (0.81-0.94) | 0.90 (0.74-1.09) | 0.81 (0.68-0.97) |

^a^ Adjusted HRs based on stratified Cox regression. Unless otherwise specified, the models were stratified for week of specimen, LTLA of residence; and used regression adjustment for date of positive test (linear), age (restricted cubic splines with 4 knots), sex, IMD rank (restricted cubic splines with 4 knots), ethnicity, vaccination status and recent international travel.
^b^ Regression adjustment for vaccination status at time of positive test, in categories: unvaccinated, <21 days since first dose, ≥21 days since first dose and <14 days since second dose, 14-55 days since second dose, 56-97 days since second dose, 98-139 days since second dose, 140-174 days since second dose, or ≥175 days since second dose. 

**Sensitivity analysis: epidemic phase bias** 

**Methods**

When comparing an outcome between cases with two different virus variants which are in different phases of incidence growth, controlling for the (observed) date of positive test rather than the (unobserved) date of infection may introduce so-called epidemic phase bias [1]. To adjust for epidemic phase bias, a sensitivity analysis was recently proposed that involves correction of the date of positive test by the assumed difference in the mean time from infection to positive test between those with and without a disease severity outcome [1].

We applied the sensitivity analysis using the primary model, based both on the entire dataset and for a subset of symptomatic and likely symptomatic cases. The Pillar 2 community mass testing programme includes testing of symptomatic individuals as well as asymptomatic individuals identified through contact tracing efforts. Cases identified through Pillar 2 self-reported whether they had COVID-19 symptoms at the time of the positive test. In addition to those who were recorded to be symptomatic at the time of their positive test, we considered all who experienced at least one of the COVID-19 severity outcomes (i.e. those who were admitted to hospital or attended emergence care within 14 days or died with COVID-19 on the death certificate within 28 days) to likely have had symptomatic disease, i.e. we assumed that they were presymptomatic at the time of their test. Symptom status was not systematically available in the hospital testing programme Pillar 1, which includes testing of patients in healthcare settings and routine testing of healthcare staff. The proportion with at least one of the outcomes was higher for those cases who were identified through Pillar 1 (6,888/56,519, 12.2%) than for symptomatic Pillar 2 cases (8,754/241,984, 3.6%) and so it may be assumed that a high proportion of Pillar 1 cases were symptomatic. Few cases in the dataset were identified through Pillar 1 (N=56,519) compared to the number of symptomatic cases identified through Pillar 2 (N=464,518), and so the inclusion of Pillar 1 cases would likely result in the inclusion many symptomatic cases, and the asymptomatic cases from Pillar 1 would only somewhat inflate the total number of cases included in the analysis. Therefore, the symptomatic and likely symptomatic subgroup included: 

1. cases from Pillar 2 with recorded symptomatic status,

2. cases from Pillar 2 who were not known to be symptomatic at the time of positive test but who experienced at least one of the COVID-19 severity outcomes (admitted to hospital or attended emergence care within 14 days or died with COVID-19 on the death certificate within 28 days), and 

3. all cases from Pillar 1. 

**Plausible values**

Time from infection to symptom onset in symptomatic cases: It has previously been estimated that the mean time from infection to symptom onset is approximately 6 days [2]. Because most symptomatic COVID-19 cases do not experience severe outcomes, this estimate may primarily be applicable to non-hospitalised and non-deceased cases. We assumed that the mean time from infection to symptom onset was likely lower for the ultimately hospitalised or deceased cases, but not less than 2 days [1].

Time from symptom onset to test: Among symptomatic Pillar 2 cases with recorded symptom onset dates, the mean time from symptom onset to positive test was 2.43 days for cases who had at least one outcome and 2.18 days for cases who experienced none of the outcomes. The corresponding mean times were similar between AY.4.2 and non-AY.4.2 Delta cases. Based on this, we assumed that the time from symptom onset to positive test did not differ between deceased and non-deceased symptomatic cases.

Hence, plausible bounds for the difference in mean time from infection to positive test between symptomatic cases without and with at least one outcome may be 0 to 4 days. For asymptomatic cases, the corresponding difference in mean time from infection to positive test is unknown.

**Results**

For all cases, and for the subgroup of symptomatic and likely symptomatic cases, the point estimates of the HR between AY.4.2 and non-AY.4.2 cases were slightly lower when the difference in mean time from infection to positive test between those without and with outcome(s) was assumed greater. The point estimates indicated HRs in the range 0.77-0.89 of hospital admission, 0.79-0.90 of hospital admission or emergency care attendance, 0.79-0.97 of COVID-19 mortality, and 0.78-0.92 of any-cause mortality. For the outcomes hospital admission, and hospital admission or emergency care attendance, the CIs excluded 1.0 and suggested slightly lower risks for AY.4.2 than non-AY.4.2 cases for all values within the plausible range of 0 to 4 days for the assumed difference in mean time between infection and positive test.

**Supplementary Table 3:** Epidemic phase bias sensitivity analysis: all cases

| **Assumed mean difference in time from infection to positive test in cases with no outcome vs cases who were admitted to hospital, attended emergency care and/or died with COVID-19 on the death certificate** | **HR (95% CI), AY.4.2 vs non-AY.4.2 Delta** ^**a**^ |  |  |  |
| --- | --- | --- | --- | --- |
|  | **Hospital admission within 14 days after specimen** | **Hospital admission or emergency care attendance within 14 days after specimen** | **COVID-19 death within 28 days after specimen** | **Death due to any cause within 28 days after specimen** |
| 0 days (no correction) | 0.85 (0.77-0.94) | 0.87 (0.81-0.94) | 0.85 (0.71-1.03) | 0.82 (0.69-0.98) |
| 1 day | 0.83 (0.74-0.91) | 0.85 (0.80-0.92) | 0.83 (0.68-1.00) | 0.80 (0.67-0.95) |
| 2 days | 0.80 (0.72-0.89) | 0.83 (0.77-0.89) | 0.81 (0.67-0.98) | 0.79 (0.66-0.94) |
| 3 days | 0.79 (0.71-0.87) | 0.81 (0.75-0.87) | 0.83 (0.69-1.00) | 0.81 (0.68-0.96) |
| 4 days | 0.77 (0.69-0.85) | 0.79 (0.73-0.85) | 0.79 (0.65-0.96) | 0.78 (0.65-0.93) |

^a^ Adjusted HRs based on stratified Cox regression. Unless otherwise specified, the models were stratified for week of specimen, LTLA of residence; and used regression adjustment for date of positive test (linear), age (restricted cubic splines with 4 knots), sex, IMD rank (restricted cubic splines with 4 knots), ethnicity, vaccination status and recent international travel.

**Supplementary Table 4:** Epidemic phase bias sensitivity analysis: symptomatic and likely symptomatic cases

| **Assumed mean difference in time from infection to positive test in cases with no outcome vs cases who were admitted to hospital, attended emergency care and/or died with COVID-19 on the death certificate** | **HR (95% CI), AY.4.2 vs non-AY.4.2 Delta** ^**a**^ |  |  |  |
| --- | --- | --- | --- | --- |
|  | **Hospital admission within 14 days after specimen** | **Hospital admission or emergency care attendance within 14 days after specimen** | **COVID-19 death within 28 days after specimen** | **Death due to any cause within 28 days after specimen** |
| 0 days (no correction) | 0.89 (0.80-0.98) | 0.90 (0.84-0.97) | 0.97 (0.80-1.17) | 0.92 (0.76-1.10) |
| 1 day | 0.87 (0.78-0.96) | 0.89 (0.83-0.95) | 0.94 (0.77-1.14) | 0.89 (0.74-1.06) |
| 2 days | 0.85 (0.76-0.94) | 0.86 (0.80-0.93) | 0.91 (0.75-1.11) | 0.88 (0.73-1.05) |
| 3 days | 0.82 (0.74-0.92) | 0.84 (0.78-0.90) | 0.89 (0.73-1.07) | 0.86 (0.72-1.03) |
| 4 days | 0.80 (0.72-0.89) | 0.82 (0.76-0.88) | 0.85 (0.70-1.03) | 0.83 (0.69-1.00) |

^a^ Adjusted HRs based on stratified Cox regression. Unless otherwise specified, the models were stratified for week of specimen, LTLA of residence; and used regression adjustment for date of positive test (linear), age (restricted cubic splines with 4 knots), sex, IMD rank (restricted cubic splines with 4 knots), ethnicity, vaccination status and recent international travel.

**References, supplementary material**

1.	Seaman SR, Nyberg T, Overton CE, Pascall D, Presanis AM, De Angelis D. Adjusting for time of infection or positive test when estimating the risk of a post-infection outcome in an epidemic. medRxiv. 2021:2021.08.13.21262014.

2.	Rai B, Shukla A, Dwivedi LK. Incubation period for COVID-19: a systematic review and meta-analysis. J Public Health. 2021.
